# Effects of physical activity on sleep in an ecologically valid design

**DOI:** 10.1093/abm/kaag025

**Published:** 2026-05-27

**Authors:** Sára Wolf, Róbert Bódizs, Péter Przemyslaw Ujma

**Affiliations:** Institute of Behavioural Sciences, Semmelweis University, Nagyvarad ter 4., Budapest H-1089, Hungary; Institute of Behavioural Sciences, Semmelweis University, Nagyvarad ter 4., Budapest H-1089, Hungary; Institute of Behavioural Sciences, Semmelweis University, Nagyvarad ter 4., Budapest H-1089, Hungary

**Keywords:** sleep, physical activity, exercise, mobile EEG, BSETS, multiday observational study

## Abstract

**Background:**

Both sleep and physical activity (PA) are essential for health. Previous studies found inconsistent effects of PA, including evening PA (EPA), on sleep.

**Purpose:**

To examine the effects of PA and its timing on objectively and subjectively measured sleep in predominantly healthy, young adults.

**Methods:**

In the Budapest Sleep, Experiences and Traits Study, a highly ecologically valid multiday observational study, 267 participants tracked their natural sleep and reported PA for at least 1 week, including mobile electroencephalography recordings. We estimated the effects of PA and its timing, quantifying it as the time elapsed between activity initiation and sleep onset.

**Results:**

Our findings showed no substantial main effect of PA on sleep (all *P* ≥ .19). However, PA temporally close to sleep had a rapid eye movement (REM) sleep-suppressing effect: each additional hour between PA and sleep onset decreased REM latency by 1.8 minutes (*B* = −1.82, SE = 0.58, *P* = .002) and increased REM percentage by 0.30 percentage points (*B* = 0.30, SE = 0.11, *P* = .008), but no other timing effect was found. Results were robust across multiple analytical specifications.

**Conclusions:**

Our results support neither a general sleep-promoting effect of PA nor a sleep-suppressing effect of EPA and suggest that for healthy young individuals, habitual, relatively low-intensity EPA is safe to perform and is a good alternative for those whose daily schedule permits no alternative timing, although the lack of detailed exercise intensity monitoring is a limitation of our study.

## Introduction

Both good sleep and regular physical activity are crucial for maintaining good health.^1^^,^^2^ Importantly, physical activity can have an influence on subsequent sleep due to the physiological changes it initiates in the body.^3^

Acute physical activity causes a general state of activation in the body, characterized by increased body temperature, blood pressure, heart rate, sympathetic activity, and cortisol levels.^4^^,^^5^ The termination of this state of activation and a homeostatic return to a more relaxed state can have a sleep-promoting effect.^6^ Medium-term physiological effects of physical activity, such as increased adenosine levels,^7^ compensatory parasympathetic activity, and the release of growth hormone, endorphins, and pro-inflammatory cytokines,^8^ may also directly promote sleep. However, if insufficient time elapses between physical activity and sleep, these longer-term sleep-promoting effects may still be overpowered by physical activity-related activation, resulting in a negative effect of late-night physical activity on sleep. In line with this two-faceted potential effect of physical activity on sleep, several recommendations from trusted sources with high search engine rankings (eg, https://www.webmd.com/sleep-disorders/ss/exercises-better-sleep, Retrieved March 18, 2025; https://sleepdoctor.com/sleep-hygiene/sleep-tips, Retrieved March 18, 2025; and https://www.hopkinsmedicine.org/health/wellness-and-prevention/exercising-for-better-sleep, Retrieved March 18, 2025) suggest engaging in physical activity to promote sleep but refraining from physical activity shortly before sleep onset. However, despite the plausibility of these recommendations, empirical research does not unequivocally support the sleep-promoting effect of daytime physical activity or the opposing effect of physical activity shortly before sleep.^9^^,^^10^

The acute effects of physical activity on sleep are generally examined in interventional studies where a group of participants is assigned to an exercise regimen, and their sleep is compared to a group of controls. The complexity and rigor of this design limits statistical power in single studies, and meta-analyses of multiple studies are preferable.

Meta-analyses of previous interventional studies examining the effects of acute and chronic physical activity on sleep have found small and heterogeneous effects^6^^,^^9-15^ (see Table S1 for an overview of meta-analytic results about physical activity effects on sleep).

Acute physical activity has been associated with modest increases in total sleep time (TST) and slow-wave sleep (SWS),^6^^,^^14^^,^^15^ and reductions in sleep onset latency (SOL)^6^^,^^13^^,^^14^ and wake after sleep onset (WASO),^14^ although the findings do not unequivocally replicate across studies. The effects on REM sleep appear more robust, with several meta-analyses reporting delayed REM onset and reduced REM duration^6^^,^^9^^,^^13-15^ following physical activity, particularly when performed in the evening or at higher intensities. However, heterogeneity across samples, variation in exercise protocols, inclusion of clinical populations, and potential publication bias limit definitive conclusions.

A large meta-analysis of experimental and interventional studies on acute physical activity,^14^ conducted on a mixed-clinical and nonclinical adult sample, found a small effect of acute physical activity on TST, sleep efficiency (SE), WASO, and N1 sleep, and a borderline significant effect on SWS and sleep quality. Earlier and newer but less inclusive meta-analyses,^6^^,^^10-12^^,^^15^ including a systematic review of randomized controlled trial (RCT) meta-analyses,^16^ found somewhat differing and heterogeneous results, including positive effects on subjective but weaker effects on objective sleep metrics. At least 2 meta-analyses have been published specifically about evening exercise,^9^^,^^13^ generally with weak to null effects, although an REM-suppressing effect was found in the most recent and inclusive one.^9^ Self-selection bias in the absence of a strict RCT protocol, heterogeneity in sample characteristics (such as the lack of separation between clinical and nonclinical samples), and uncontrolled multiple testing or publication bias limit the degree to which definitive conclusions can be drawn based on current meta-analyses.^16^ Thus, despite the considerable volume of the published literature, the specific effects of physical activity on sleep have not been unequivocally described.

An alternative to large RCTs or meta-analyses thereof are multiday observational studies,^17^^,^^18^ in which continuous monitoring of participants takes place over multiple days and nights, and the day-to-day covariation of daily experiences and subsequent sleep is studied. We previously argued^19^^,^^20^ for the utility of this design in sleep research. Multiday observational studies enable higher statistical power due to the ease of recruitment, they are highly ecologically valid as instead of interventions the variance of wake experiences comes from the normal day-to-day variability of the participants’ lives, while time-lagged, within-participant analyses control for time-invariant confounders (eg, demographic characteristics) and reverse causality, and can falsify causal hypotheses, although full causal inference is limited by the possibility of time-variant confounding.^20^^,^^21^

Some recent studies used such naturalistic designs instead of interventions to investigate the effects of physical activity on subsequent sleep. Zapalac et al^22^ studied the effects of physical activity on subsequent sleep in 65 university students using actigraphy. They found the most robust effects on sleep composition: REM latency and non-rapid eye movement/rapid eye movement (NREM/REM) ratio were increased after low-intensity physical activity but reduced after sedentary days. Sustained high-intensity physical activity was also associated with reduced SOL and higher self-reported sleep quality. Li et al^23^ studied 113 African pastoralists with actigraphy and found that TST and fragmentation were reduced by physical activity, while a positive effect on WASO and SE was exclusive to low-intensity activity. Detailed sleep composition was not studied. Seol et al^24^ in a study of 92 elderly adults with actigraphy during the day and polysomnography (PSG) during the night found that sedentary activity increased and physical activity decreased REM sleep propensity, with an opposite effect on sleep depth in some analyses. Negele et al^25^ in a study of 1223 adolescents wearing actigraphs found some effects of physical activity on SE and SOL, but these did not generalize across activity types, sexes, and days of the week, also with a limited role of activity timing relative to sleep. Detailed sleep composition and REM sleep were not studied. However, a recent study^26^ of a very large database of over 4 million nights from close to 15 000 individuals using a wrist-worn biometric device reported a dose-dependent negative effect of late-night physical activity on sleep duration and sleep percentage, with little effect of light but a substantial effect of high-intensity physical activity. Finally, Miller et al^27^ used data from a wristband from a single night to predict sleep quality from the characteristics of participants and the physical activity they undertook during the previous day. While the study focused on the automatic classification of disturbed and undisturbed sleep and not the identification of specific characteristics influencing sleep, the better-than-random performance of the authors’ model implies at least some physical activity effects. Notably, only one of the multiday observational studies^24^ used electroencephalography (EEG) to measure sleep.

In sum, observational studies are also inconclusive about the effect of physical activity on sleep. Although not always studied, a REM-suppressive effect of physical activity, also evident from the interventional literature, appears to be the best replicated finding. However, most observational studies rely on actigraphy or self-reported sleep measures, rather than EEG. The use of mobile EEG measures offers a methodological alternative to laboratory PSG and has been validated^28^^,^^29^ against PSG and demonstrated satisfactory results for measuring sleep parameters. Despite this, very few studies have examined the association between physical activity and sleep using EEG-based measures in natural settings, and we aimed to address this gap in research.

Our study aimed to investigate the effects of physical activity and its timing on objectively and subjectively measured sleep and to replicate and extend these findings in a large multiday observational study while also assessing interindividual differences in these effects. The novelty and strength of our study is the use of mobile EEG measures. Actigraphy is suitable for observational studies as it offers an alternative to laboratory testing of sleep; however, these devices only provide indirect estimates of sleep-wake patterns based on body movement and cannot distinguish sleep stages and REM reliably.^30^^,^^31^ In contrast, EEG devices can directly measure electrical brain activity and remain the gold standard for sleep staging, allowing more precise characterization of sleep architecture. EEG also uniquely enables the assessment of sleep-specific oscillations, including SWS, even though this is crucial to mechanistically understand the effect of physical activity. SWS is a sensitive indicator of sleep pressure, which is homeostatically upregulated during wakefulness, with a hypothetical additional upregulation due to increased daytime activity,^32^ including, hypothetically, physical activity.^33^

Our hypothesis was that physical activity would have minimal effects on sleep parameters in a healthy population and that physical activity performed closer to sleep onset would be associated with suppressed REM sleep without major sleep disruption.

## Methods

### Study design

Our study used a multiday, time-lagged, within-participant observational design. Participants were monitored in their natural environment for at least 1 week and during recorded self-reports about their daily activities and experiences and measured sleep objectively by mobile EEG headbands.

### Participants

Our analysis was performed with data from the Budapest Sleep, Experiences and Traits Study (BSETS). The full BSETS protocol has been published elsewhere.^19^ Briefly, BSETS participants were healthy volunteers who, for a period of at least 1 week, recorded their daily experiences in an evening diary and their sleep using a Dreem2 mobile EEG device and completed a morning diary about subjective nightly experiences. Participants were recruited using branched diffusive convenience sampling, mostly from students of Semmelweis University and their relatives. As maximal ecological validity was a key goal, inclusion criteria were minimal and consisted only of being at least 18 years of age, being fluent in Hungarian, and not being recruited from clinical settings. BSETS was designed to maximize ecological validity by using time-lagged, within-participant data to identify the naturally occurring daily experiences (eg, affective experiences, physical and mental exhaustion, sexual activity—in-depth analyses of the indicators we used are published elsewhere^34^) that are followed by alterations in sleep (and vice versa). Consequently, participants received no intervention and were free to schedule their days and sleep as they saw fit, as long as it was faithfully recorded.

The Institutional Review Board (IRB) of Semmelweis University, as well as the Hungarian Medical Council (under 7040-7/2021/EÜIG “Vonások és napi események hatása az alvási EEG-re” [The effect of traits and daily activities and experiences on the sleep EEG]), approved BSETS as compliant with the latest revision of the Declaration of Helsinki. All participants gave written informed consent on a form reviewed and approved by the IRB.

### Data collection

Data collection was performed between May 2021 and April 2023.

### Physical activity

BSETS participants indicated in each evening diary if they exercised on that day as a binary variable. The questionnaire requested whether the participant has performed any kind of “exercise” for at least 30 minutes during the day and then asked about the specific type of physical activity the person regarded as “exercise.” This way, we have assessed physical activity based on self-reporting, and consequently, physical activity included any activity that the participant classified as such. Due to the nature of this free-form data collection, reported activities included a heterogeneous mix of leisure-time and lifestyle physical activities (eg, running, gym workouts, cycling, and walking); consequently, these were not systematically categorized due to variability in reporting detail. An additional free-form diary was completed in the same evening questionnaire, from which the timing of “exercise” could be recovered. Information on the intensity and duration of the reported physical activity was not systematically collected.

Out of 1792 days with valid data, we recorded 577 days with indicated physical activity. The intraclass correlation (estimated here as the Nagelkerke *R*^2^ of a logistic regression model using physical activity as a binary outcome and participant ID as the sole categorical predictor) was 0.22, indicating that physical activity was moderately clustered within individuals. The mean participant indicated physical activity on 32.14% of study days (SD = 27%). Out of 257 participants with physical activity data, 60 participants recorded zero days with physical activity and 5 recorded physical activity each day, making them uninformative for within-person analyses, which make use of the 191 participants who indicated physical activity on some but not all days.

For 333 days, the timing of physical activity initiation could be recovered from the evening diaries. Sleep onset was available from nightly EEG recordings on 1718 nights. Whenever possible from this data (*N* = 314 days), we calculated the amount of time elapsed from the start of physical activity until EEG-detected sleep onset, expressed in hours.

Physical activity termination, although a more straightforward expression of physical activity timing for our purposes, was reported less frequently (*N* = 231 days, of which *N* = 227 also reported physical activity initiation), and it was highly correlated with physical activity initiation (*r* = 0.95). Therefore, we decided to use physical activity initiation as the key variable to express the relative timing of physical activity and sleep.

### Sleep recording

In our observational study design, participants followed their habitual sleep schedules, with no prescribed bedtimes or wake times. The BSETS protocols included both EEG-based objective and diary-based subjective sleep monitoring. A set of sleep variables was extracted from both as outcomes of interest that can be affected by previous physical activity.

During the night, participants recorded their sleep using a Dreem2 mobile EEG headband.^35^ This headband uses 5 dry silicon electrodes with a sampling frequency of 250 Hz. The Dreem2 electrodes approximate standard 10-20 system locations (F7, F8, Fpz, O1, and O2), although exact placement is optimized for a wearable dry-electrode configuration. Figure S1 shows the device structure and its electrode placement relative to the 10-20 system. Sleep was scored automatically using a validated proprietary algorithm,^28^ and sleep macrostructure indicators, such as sleep onset timing, SE, TST, SOL, REM latency, WASO, the duration and percentage of N1, N2, N3, and REM sleep, as well as the number of awakenings, were extracted as variables of interest. The algorithm also provided information on mean respiration frequency.

In line with previous BSETS analyses,^17^^,^^19^^,^^20^ we calculated power spectral density (PSD) in the delta (0.5-4 Hz) and low sigma (10-13 Hz) ranges from the channel F7-O1 to track slow wave and sleep spindle activity, respectively. PSD was calculated using the periodogram() MATLAB EEGLab function with 2-second nonoverlapping epochs and Hamming windows. The choice of this channel was motivated by a good trade-off between data quality and good topographical mapping of widespread activities, and a focus on the low rather than the fast spindle range was motivated by the frontal location of Dreem2 electrodes.^19^

In addition to objectively recorded data, we also used subjective sleep quality reported in the morning using the Hungarian version of the Groningen Sleep Quality Scale (GSQS)^36^ as an outcome of interest. The 15-item self-report questionnaire assesses perceived sleep quality—the difficulty of falling asleep and sleep disturbances during the previous night. Higher scores indicate poorer subjective sleep quality. The GSQS has demonstrated good internal consistency and validity in both clinical and nonclinical samples.

### Heart rate detection

The use of the Dreem2 mobile EEG device allowed us to calculate heart rate (HR) and its variability during the night. Dreem2 records cardiac activity using an infrared pulse oximeter,^35^ but heart rate and its variability are not processed by the device’s proprietary algorithm. Therefore, we implemented a custom algorithm to process raw pulse oximeter data. The raw signal was first band-pass filtered to 0.5-3 Hz, allowing for heart rates in the range of 30-180 beats per minute. Then, the instantaneous signal phase was computed by calculating the phase angle of its Hilbert transform. Our assumption was that unusually large phase differences will indicate the termination of a cardiac cycle and can serve as the basis for determining heart rate. To accomplish this, we first calculated the differentials of phase angles and then the histogram thereof. As expected, most phase differentials were small positive numbers, with a substantial minority (∼1.5%, corresponding to a heart rate of ∼60/minute) in the [−6.28 to −6] range, corresponding to a decrease of about 2 radians or a full phase reset. In order to precisely determine the threshold below which a negative phase difference could be identified as the termination of a cardiac cycle, we modeled the histogram as a combination of 2 Gaussian distributions (with a Gaussian mixture model using 2 distributions, implemented with the fitgmdist() MATLAB function). The detection threshold was set where the probability distributions of the 2 fitted Gaussians intersected, that is, below which value it was more likely that a value came from the distribution of phase resets and not the distribution of ordinary phase changes from within the cardiac cycle. Figure S2 illustrates this process.

The instantaneous heart rate was calculated for each detected peak. Abnormally short (<0.33 seconds, implying HR >180) or abnormally long (>2 seconds, implying HR < 30) heartbeats were deleted. Cleaned heart rates were averaged for wake periods (to serve as a baseline), as well as separately for the first hour of recordings (to capture the immediate effects of physical activity). Standard deviations instead of averages were also saved to express heart rate (more precisely, pulse) variability. We note that while our method enabled calculating heart rate over longer periods, heart rate/pulse variability, which requires a more precise detection of individual heartbeats, can only be calculated with limitations using the low-frequency signal available from the pulse oximeter. While our algorithm has not been independently validated, its output showed physiologically plausible heart rate distributions and sensitivity to physical activity and was consistent across nights within individuals. We therefore interpret heart rate variability results cautiously and emphasize within-participant differences rather than absolute values.

### Statistical analysis

BSETS recorded physical activity and sleep from the same participants over several consecutive days with a known temporal order of the 2. In such a design, the time-lagged, within-participant regression coefficients estimate how deviations in an individual’s physical activity from their own average are associated with subsequent deviations in their sleep parameters, thereby providing causally informative estimates of the within-person effects of physical activity on sleep. Causal inference from this design has the limitation that some confounding (not physical activity, but the causes or consequences of physical activity being the cause of sleep alterations) is still possible. However, by design, all time-invariant individual characteristics (eg, age, sex, personality traits) are fully controlled in within-participant analyses, and because of its time-lagged nature, the design excludes reverse causality, and it is well-suited to falsifying causal hypotheses.^20^^,^^21^ In addition to age, sex, and day of the week, our model controlled for the previous night’s value of the dependent sleep parameter. Other time-varying confounders such as caffeine intake and alcohol consumption were not systematically controlled for; however, previous analyses suggest that the contribution of such factors to within-person sleep variability is modest.^34^

As an observational study, all analyses treated physiological and sleep-related measures as dependent variables. These included sleep architecture measures of the mobile EEG device (eg, TST, SE, REM, and NREM stage proportions) and its spectral power, as well as subjective sleep quality ratings and nocturnal cardiovascular parameters (rate and variability) and respiratory rate.

In our analyses, we modeled sleep parameters in the following way:


$$\begin{array}{c} \text{sleep}∼\text{physical activity}\_\text{means}+\text{physical activity}\_\text{deviations}\\ +\text{timing}+\text{lagged}\_\text{sleep}+\text{age}+\text{sex}+\text{weekend}+\left(\text{1}\text{ID}\right) \end{array}$$

That is, in a mixed-effects model, we expressed each sleep ­parameter as a function of:

1. the proportion of “exercise” days in each individual (between-participant effects),
2. whether the participant indicated physical activity before the given night, as a binary variable (within-participant effect),
3. physical activity timing (the time difference between physical activity and sleep onset, in hours),
4. the sleep parameter from the previous night,
5. age, sex, and day of the week (weekday/weekend) as controls, and
6. a random intercept per participant.

Note that this model estimated the main effect of physical activity (which requires both “exercise” and “non-exercise” days) and the effect of physical activity timing (which is only interpretable for “exercise” days) simultaneously. This issue was solved by centering “timing” at the mean value (14:50:24) and entering this centered 0 value for “non-exercise” days. This ensured that while only cases with meaningful timing values informed the analyses about timing, “non-exercise” days were still available to estimate the main effect of physical activity. It also ensured that the effect of “timing,” although formally a main effect term, captures the interaction between physical activity and physical activity timing, that is, how much the effect of physical activity on sleep is expected to change if more time elapses between physical activity and sleep. This is because a formal interaction term, “physical activity * timing” is identical to “timing” expressed this way: it is zero on days with no indicated physical activity and identical to the actual timing on “exercise” days.

Due to the need to know of physical activity timing in these models, we dropped cases from analyses if physical activity was reported but its timing was not recoverable from evening diaries. This way, analyses involving physical activity timing were conducted as complete-case analyses, excluding days with missing timing information. Because this may result in bias, in additional analyses (see *Results* section), we also calculated the main effect of physical activity without timing interactions and using all cases.

The most important parameters from our model were “physical activity deviations” and “timing.” “Physical activity deviations” represent the estimated within-person change in the sleep parameter as a function of physical activity, indicating changes relative to whether a participant indicated physical activity on a given day. “Timing” refers to the estimated change in this effect per one additional elapsed hour between physical activity and sleep.

In addition to calculating time elapsed between physical activity and sleep based on the time of starting the reported physical activity, we have also estimated the timing interaction using the time of the physical activity midpoint and end. From daily diaries, we could recover the time of physical activity start for 57.7% of “exercise” days. Although less data (40% for the end, 39.4% for the midpoint) is available this way, the end or midpoint of physical activity is arguably a better indicator of its sleep-influencing effects. Additionally, in another alternative analysis, instead of the time elapsed between physical activity and sleep, we considered the clock time of physical activity.

Based on the content of the daily diaries, we could assign an approximate physical activity timing (“morning” before 12:00, “afternoon” between 12:00 and 18:00, and “evening” after 18:00) to 133 further “exercise” days for which this could not be recovered with hourly precision (*N* = 466 in total, 80.77% of all “exercise” days). We re-ran our analyses using this categorical timing system, where physical activity timing was used as a single categorical predictor (other than the usual controls) of sleep variables, with “no physical activity” as the reference category and pairwise contrasts between the 3 physical activity timings. Within- and between-participant effects were not separated, as these were not interpretable for this variable. In this model, 3 different main effects of physical activity (one for each timing category) are simultaneously estimated, while contrasts (eg, significantly greater changes in sleep after evening than morning physical activity) are analogous to interaction effects.

In further sensitivity analyses, we used self-reported physical exhaustion during the day instead of self-reported physical activity as an alternative indicator of physical strain with a potential effect on sleep.

As in previous BSETS papers,^17^^,^^20^^,^^37^ we used 2 significance thresholds to identify putatively and confidently significant effects while correcting for multiple comparisons. We considered effects putatively significant if the *P-*value remained at <.01. We also performed a formal control for false discovery rates (FDR) using the Benjamini-Hochberg method,^38^ conducted on the pool of all investigated physiological parameters.

## Results

### Descriptive statistics

A total of 267 participants took part in BSETS, of whom hypnogram data were available from 258 (due to missing EEG recordings from the rest) and quantitative EEG data from 249 (due to the lack of sufficient data quality in 9). Some missingness in the data was present. See Table 1 for precise variable-wise sample sizes. See Figure S3 for a flow diagram summarizing exclusion and inclusion in our studied population.

**Table 1:** Descriptive statistics of key study variables, showing univariate sample sizes.

	Valid sample size (*N*)	Mean	Median	SD	ICC
**Total sleep time**	1712	396.85	403.00	91.12	0.24
**Sleep onset latency**	1712	14.86	10.50	14.71	0.31
**REM latency**	1706	79.32	70.50	36.77	0.23
**Sleep onset time**	1712	0.43	0.17	1.79	0.49
**Wake after sleep onset**	1712	21.05	15.00	21.59	0.38
**Sleep efficiency**	1712	91.05	92.79	6.71	0.36
**N1 %**	1712	6.08	5.59	2.49	0.52
**N2 %**	1712	46.30	46.40	9.18	0.46
**N3 %**	1712	21.78	21.07	9.50	0.48
**REM %**	1712	25.84	25.46	7.49	0.30
**N1 duration**	1712	24.05	22.00	10.91	0.53
**N2 duration**	1712	186.24	185.50	60.73	0.35
**N3 duration**	1712	82.97	84.00	31.90	0.58
**REM duration**	1712	103.59	101.50	38.48	0.25
**Awakenings**	1712	19.59	18.00	9.47	0.51
**Self-rated sleep**	1745	4.13	4.00	3.41	0.20
**Delta power**	1453	1.12	1.11	0.19	0.56
**Sigma power**	1453	−0.16	−0.16	0.14	0.72
**Physical activity**	1792	0.32			0.46
**Time from physical activity to sleep**	1525	0.00	0.00	1.90	0.17
**Physical activity clock time**	1546	0.00	0.00	2.09	0.25
**First hour heart rate**	1506	63.15	62.41	9.04	0.53
**Wake heart rate**	1505	64.55	63.95	7.20	0.49
**Respiration rate**	1712	15.38	15.53	2.99	0.92
**Wakefulness duration**	1407	17.07	16.88	1.96	0.12

The sample sizes used in the main models are shown in Figure S3, described with exclusion criteria by a flowchart. Duration variables are expressed in minutes, except for wakefulness duration, which refers to wakefulness between the previous and current’ night’s sleep and is expressed in hours. Self-rated sleep refers to raw scores on the Groningen Sleep Quality Scale. EEG power is expressed in log units. Time from physical activity to sleep, as well as physical activity clock time are scaled so that the average value (8.7 hours for time elapsed and 14:50:24 for clock time) is zero but the units are retained as hours.

Abbreviations: ICC, intraclass correlation coefficient; REM, rapid eye movement.

Of the participants, 45% were male and 55% female. The mean age was 28.86 years (SD = 12.73 years, range: 18-76 years). The age distribution of the sample was highly skewed, with 64.4% of the sample being younger than 25 years old, 10.7% being 26–35 years old, 5.1% 36–45 years old, 16.2% 46–55 years old, and 3.6% older than 55 years old. This age distribution reflects the fact that most participants were friends or parents of medical students. In addition, 58.8% were university students; 51.4% were single, 24.5% had a partner, 21.4% were married, 2.33% were divorced, and one participant was widowed. As an indicator of socioeconomic status, we assessed the participants’ highest level of education. The majority had a high school diploma (51.8%). A further 17.5% obtained a university master’s degree, 12.1% a university bachelor’s degree, 6.6% a college degree, and 3.9% reported a higher qualification. In addition, 5.1% had completed primary education only (8 years), 1.9% had graduated from a vocational secondary school with a diploma, and 1.2% had a vocational school certificate. These educational characteristics are consistent with the age distribution of the sample, reflecting that many participants were still enrolled in university studies. We did not collect data regarding participants’ ethnicity.

Descriptive variables, including valid sample sizes and intraclass correlation coefficients, are reported in Table 1.

### Main analyses

In our main analyses, we estimated both the main effects of physical activity (differences in sleep parameters and cardiac/respiratory activity after physical activity) and their interaction with physical activity timing (changes in the main effects with increasing temporal distance between physical activity and sleep). Within-participant effects were of primary importance, as these indicate putative causal effects of physical activity on sleep (see *Methods* for limitations on causal inference). However, we also calculated between-participant effects reflecting the cross-sectional relationship between physical activity habits and typical sleep parameters.

Physical activity had no significant within-participant main effect on any sleep or physiological parameter (*P*_min_ = .19). Physical activity more adjacent to sleep, however, had a REM-suppressing effect. Specifically, for each additional hour that elapsed between physical activity and sleep, REM latency was reduced by 1.8 minutes (*B* = −1.82, SE = 0.581, *P* = .002, significant after FDR correction), and REM percentage increased by 0.3 percentage points (*B* = 0.299, SE = 0.112, *P* = .008, not significant after FDR correction).

Between-participant effects showed reduced heart rate, N1 sleep, and EEG sigma power, but increased sleep time in those who indicated physical activity frequently. Habitually exercising closer to sleep was associated with better self-reported sleep quality.

**Figure 1:**
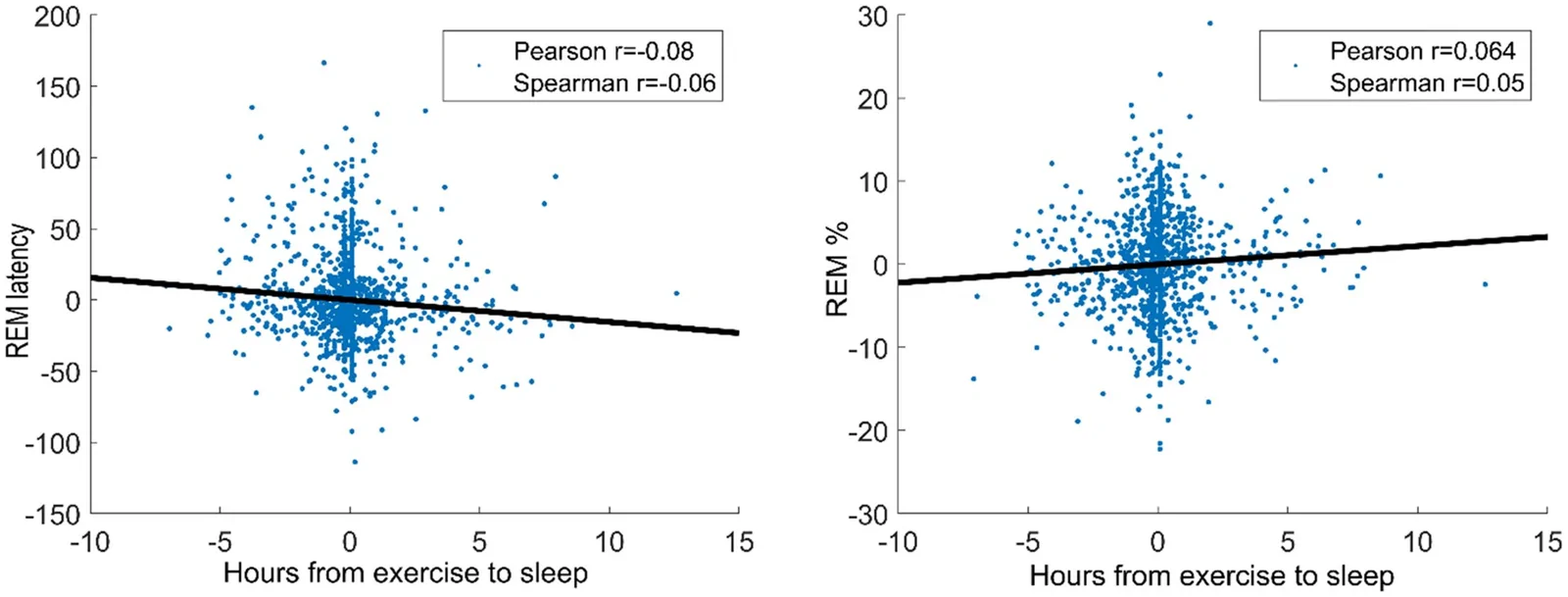
Late-night physical activity suppresses REM sleep. Less time between physical activity and subsequent sleep was associated with increased REM latency (left, significant after FDR correction) and reduced REM percentage (right, not significant after FDR correction). The scatterplots show deviations from the individual means of sleep parameters and physical activity timing, residualized for age, sex, and day of the week. A best-fit OLS regression line, Pearson and Spearman correlations of the residualized deviations are shown. Abbreviation: FDR, false discovery rate; OLS, ordinary least squares; REM, rapid eye movement.

Table 2 summarizes findings in detail. Figure 1 illustrates the REM-suppressing effect of late-night physical activity.

**Table 2:** The effects of physical activity and its timing on sleep variables.

	Physical activity main effects						Timing interactions					
	Within-participant			Between-participant			Within-participant			Between-participant		
	*B*	SE	*P*	*B*	SE	*P*	*B*	SE	*P*	*B*	SE	*P*
**Total sleep time**	−7.874	7.002	0.261	**30.528**	**12.210**	**0.013**	−0.987	1.439	0.493	−6.324	2.938	0.032
**Sleep onset latency**	−0.020	0.023	0.385	0.105	0.052	0.043	0.004	0.005	0.385	0.005	0.012	0.714
**REM latency**	2.930	2.839	0.302	7.869	5.214	0.132	−**1.818**	**0.581**	**0.002**	−1.711	1.252	0.172
**Wake after sleep onset**	−0.015	0.023	0.528	−0.069	0.050	0.166	0.003	0.005	0.547	0.000	0.012	0.967
**Sleep efficiency**	−0.011	0.018	0.554	−0.028	0.044	0.517	0.002	0.004	0.595	0.012	0.010	0.248
**N1 %**	0.095	0.163	0.560	−**0.934**	**0.370**	**0.012**	0.044	0.033	0.186	0.098	0.088	0.264
**N2 %**	0.125	0.607	0.837	1.112	1.339	0.407	−0.242	0.123	0.049	−0.521	0.320	0.103
**N3 %**	0.101	0.636	0.873	0.445	1.244	0.721	−0.091	0.130	0.485	0.318	0.298	0.286
**REM %**	−0.219	0.544	0.687	−0.562	0.944	0.552	*0.299*	*0.112*	*0.008*	0.007	0.228	0.975
**N1 duration**	−0.128	0.705	0.856	−2.076	1.745	0.234	−0.021	0.142	0.881	−0.014	0.414	0.974
**N2 duration**	−3.828	4.327	0.377	20.461	9.139	0.025	−1.373	0.879	0.119	−4.617	2.181	0.034
**N3 duration**	−0.378	1.939	0.845	7.848	5.046	0.120	−0.351	0.390	0.369	−0.343	1.195	0.774
**REM duration**	−3.923	2.995	0.190	3.613	4.902	0.461	0.813	0.618	0.189	−2.032	1.187	0.087
**Awakenings**	−0.732	0.627	0.243	−3.235	1.620	0.046	−0.004	0.126	0.972	0.057	0.384	0.881
**Subjective sleep quality (GSQS)**	0.293	0.267	0.273	0.376	0.444	0.398	0.094	0.054	0.084	**0.383**	**0.108**	**<0.001**
**Delta power**	−0.006	0.013	0.664	−0.011	0.027	0.677	0.002	0.003	0.520	0.002	0.007	0.787
**Sigma power**	−0.006	0.009	0.476	−**0.032**	**0.012**	**0.008**	0.001	0.002	0.689	0.000	0.003	0.956
**Respiration rate**	−0.006	0.086	0.947	0.129	0.114	0.256	0.017	0.018	0.358	−0.023	0.028	0.422
**Heart rate (wake)**	0.540	0.433	0.213	−**4.089**	**1.231**	**0.001**	−0.113	0.087	0.191	−0.018	0.309	0.954
**Heart rate variability (wake)**	−3.600	7.910	0.649	−19.915	18.861	0.291	0.591	1.597	0.711	−2.609	4.729	0.581
**Heart rate (1st hour)**	0.890	0.605	0.141	−**5.535**	**1.445**	**<0.001**	−0.178	0.122	0.145	−0.126	0.359	0.725
**Heart rate variability (1st hour)**	10.761	12.291	0.382	−**73.511**	**25.759**	**0.004**	−1.739	2.502	0.487	−4.850	6.412	0.450

Regression coefficients indicate the estimated changes in sleep parameters after physical activity (main effects), or changes in these effects per 1 hour of additional distance between physical activity and sleep (interactions). Within-participant effects indicate sleep changes within the same participant after indicated physical activity, while between-participant effects are correlations of physical activity habits and average sleep. All regression coefficients are expressed in raw units, and in minutes in case of timing variables. Bold values indicate effects which survive correction for multiple comparisons. Values in italics do not survive this correction but are putatively significant at *P* < .01.

Abbreviation: REM, rapid eye movement.

### Alternative physical activity timing specifications

In alternative analyses using physical activity end or midpoint, we could replicate our main findings of no within-participant main effects and sleep-adjacent physical activity extending REM latency, although the REM percentage-reducing effect was reduced to a trend. While lower heart rate in more frequent exercisers was replicated, lower subjective sleep quality in early exercisers was not. Detailed model output is available in Table S2 (midpoint) and Table S3 (end).

When considering the clock time of physical activity, REM-suppressive effects of late-night physical activity on latency (*P* = .004) and proportion (*P* = .035) were reduced to trends, in line with time relative to sleep, not raw timing itself, being of primary importance. Findings are summarized in Table S4.

### Categorical physical activity timing

We re-ran analyses using a categorical timing variable instead of the continuous one previously used. No significant effects of physical activity on sleep or physiological parameters, and no significant contrasts emerged. Detailed results are reported in Table S5.

### Only physical activity main effects

In previous analyses, we simultaneously considered the main effect of physical activity on sleep, as well as its interaction with timing. For these analyses, it was necessary to exclude cases with valid physical activity data but no recoverable timing. This reduces statistical power to detect the main effect of physical activity and may bias analyses if timing is missing non-randomly. Therefore, we re-calculated models without timing data, focusing only on the main effect of the binary physical activity variable. As in the main analyses, physical activity had no significant effect on any sleep or physiological parameters, although the positive effect on first hour and wake heart rate, as well as first-hour heart rate variability, approached nominal significance (*P* = .07-0.13). Detailed results are reported in Table S6.

### Physically exhausting days

BSETS participants described the previous day as “Physically exhausting” in each evening diary on a Likert scale ranging from 0 to 10. The mean level of physical exhaustion was reported at 4.45 (SD = 2.44). After days with self-reported physical activity, physical exhaustion was reported as 1.83 points higher (SE = 0.11, *P* = 10^−56^, from a mixed-effects model with random intercepts per participant, incremental *R*^2^ = 13% over an intercept-only model). Physically exhausting days were followed by elevated first-hour heart rate (*B* = 0.458 BPM/Likert point, *P* < .001), a stronger effect than for physical activity itself. No significant sleep changes were detected, however, after more physically exhausting days. Detailed findings are reported in Table S7.

## Discussion

In our large, ecologically valid study of predominantly healthy young adults, we show evidence from a mobile EEG database that physical strain, including evening physical activity, has limited effects on sleep. Our findings are in line with other ecologically valid observational studies,^22^^,^^24^ which found that REM suppression is the primary consequence of physical activity, with little consistent effect on other sleep parameters.^25^ Across multiple model specifications (including models using different physical activity timing variables and a standalone main analysis of physical activity effects), self-reported physical activity or physical exhaustion had no main effect on any sleep variable, either in the negative or positive direction. Physical activity timing, however, exhibited a REM-suppressive effect, with increased REM latency and reduced REM percentage after later physical activity, a finding that persisted across multiple model specifications. The effect on REM latency was homogeneous across participants, but the effect on REM percentage was heterogeneous. Importantly, the temporal proximity of physical activity to sleep had a stronger moderating effect than its clock time (Table 2, Table S4), suggesting that the time-dependent state of physiological renormalization following exercise determines its effect on sleep.

Meta-analyses of the effects of acute physical activity^6^^,^^14^^,^^15^ as well as late-night physical activity specifically^9^^,^^13^ agree on a REM-suppressive effect of physical activity, which is consistent with our findings. Other findings, however, appear less consistently across meta-analyses—such as improved SE, increases in TST and SWS, as well as reductions in SOL—and were not observed in our sample.

There are several hypothetical mechanisms that may make REM sleep especially sensitive to physical activity. First, it is possible that REM sleep is especially sensitive to some physiological changes caused by physical activity, such as elevated sympathetic dominance, adrenergic and noradrenergic activity, or increases in body temperature,^9^^,^^39^^,^^40^ leading to a longer time window before physiological renormalization is complete. Additionally, the physiological changes from physical activity to sleep may also be influenced by behaviors occurring in between the two. Activities such as eating, hydration, showering, or screen use may modulate the rate of physiological renormalization, potentially modulating subsequent sleep architecture as well. Although in our current study these factors were not systematically assessed in relation to physical activity, it is an important target for future research.

Second, homeostatic pressure for sleep may be higher after physical activity^41^^,^^42^ and may favor NREM to REM sleep, as physical activity may increase cerebral adenosine accumulation, although this is contradicted by the lack of a compensatory NREM increase in our study. Third, physical activity may act as a zeitgeber and disrupt the circadian rhythm, affecting REM regulation, which is strongly tied to the circadian rhythm.^43^

A possible explanation for heterogeneous non-REM effects in interventional studies that do not replicate in more ecologically valid observational designs is a difference in the physiological burden of physical activity. The speed of sympathetic withdrawal and parasympathetic reactivation following physical activity, indicated by a renormalization of heart rate and its variability, depends on physical activity intensity and fitness. Most self-directed physical activity is low or moderate in intensity^26^ and a degree of habituation is expected. In contrast, exercise regimens assigned in an interventional study are likely novel and may be more challenging, especially in clinical samples, which are often included in meta-analyses but not multiday observational studies including ours. Physiological recovery following physical activity may be fast enough to avoid impacting sleep in fit individuals engaging in familiar, low-intensity physical activity, but insufficiently so if the physical activity is novel or high-intensity given a participant’s fitness. The former may be the case in observational studies, and the latter in interventional studies, especially in clinical samples. This hypothesis is supported by the very large recent observational study of Leota et al,^26^ which found that reductions in sleep duration and quality were specific to late-night, high-intensity physical activity.

As both physical activity and sleep are crucial for maintaining good health, it is essential to explore if trade-offs exist between the two. Sleep hygiene recommendations often discourage evening physical activity, raising concerns about sleep disruption. However, our findings support recommendations (eg, https://www.sleepfoundation.org/physical-activity/best-time-of-day-to-exercise-for-sleep) that physical activity is generally safe to perform in the evening hours for healthy young adults with a low expected sleep-disrupting effect. Based on our results, no substantial negative effect on sleep is expected in individuals choosing to perform their habitual physical activity regime in the late evening hours, although this statement may not generalize to novel, high-intensity exercise regimens. These findings may reduce perceived barriers to physical activity adherence, particularly among people with strict daily schedules, and highlight the importance of observational study design over laboratory-based findings.

Our study has limitations. First, while a strength of our study was its mobile EEG paradigm, which enabled a more fine-grained analysis of sleep than most wearables, we relied on self-reports to detect physical activity and its timing. This also limited our ability to assess the intensity of physical activity. Consequently, as it was not measured in metabolic equivalents, we used the phrase “high-intensity physical activity” where necessary instead of “vigorous physical activity” to avoid confusion with the more precise terminology. Second, the majority of our sample consisted of young, healthy, relatively fit participants (mean BMI = 23.1, SD = 3.7, with over 75% recording at least one physical activity over the study week). Therefore, while the findings are informative for the healthy working-age population, where the potential negative impact of late-night physical activity on sleep is the greatest concern, they are less generalizable to clinical populations or specifically to clinical populations where sleep complaints are more common. Third, as described in detail in the *Statistical analysis* section, although our time-lagged, within-participant design strengthens causal interpretation by controlling for time-invariant confounders and excluding reverse causality, some residual confounding may still be present due to time-varying factors (the causes or consequences of physical activity) and explain part of the observed associations. Fourth, while our study lacks sufficiently precise data on physical activity intensity, most physical activity in our sample was likely relatively low-intensity and habitual, limiting our conclusion about a null effect to such physical activity and not novel, high-intensity regimens. Recent evidence^26^ suggests that despite findings of a previous meta-analysis,^9^ high-intensity late-night physical activity may negatively impact sleep.

## Conclusions

In sum, our study provides evidence that physical activity does not severely impact sleep, albeit a REM-suppressive effect is observed. The findings suggest that at least habitual, relatively low-intensity physical activity has no negative impact on sleep and should be a viable alternative to those with no other option to time their physical activity.

## Supplementary Material

kaag025_Supplementary_Data

## Transparency statements

For each of the 5 transparency practices, you will provide a statement that accurately describes your article. The following examples may be adapted for use. If none of the statements accurately describe your article, please provide a statement. **Statements should be provided on the cover page and will be published with your manuscript.** 

1. **Study registration**. Study registration involves declaring the study design, variables, and treatment conditions in an independent, institutional repository*(e.g.,*  http://clinicaltrials.gov, https://osf.io/, https://www.socialscienceregistry.org/*)*. A study is considered *pre*-registered if it is registered in a repository prior to the study began. Consider the following examples:
  - The study was pre-registered at ___________________________________ *(insert name of repository and link to study registration).*
  - This study was registered after the study began. The study is registered at ________________________________ (*insert name of repository and link to study registration*).
  - **This study was not formally registered.** 
2. **Analytic plan pre-registration**. Pre-registration of an analytic plan involves specification of the planned sequence of analyses or the statistical model that will be reported. Reporting the planned outcome variables alone does not qualify as analytic plan pre-registration. Consider the following examples:
  - The analysis plan was registered prior to beginning data collection at ____________ (*insert name of repository and link to study registration*).
  - **The analysis plan was not formally pre-registered.** 
3. **Data availability:** Indicate whether the data underlying the presented analyses will be made available in a third-party archive/repository. If data is available in an archive, indicate if this is fully available to the public or has protected access (available for limited audience, with clearly defined requirements and steps to obtain data). If data is not in a public archive, describe any steps needed to access the data. Consider the following examples:
  - **De-identified data from this study are available in a public archive:**  https://osf.io/hyntr/
  - De-identified data from this study are available in a protected archive: _________________________ (*provide url*). Data can be obtained by _________________ (*briefly describe steps and requirements to access data*)
  - De-identified data from this study are not available in an a public archive. De-identified data from this study will be made available (as allowable according to institutional IRB standards) by emailing the corresponding author.
4. **Analytic code availability.** Analytic code refers to the programming code used to conduct the analyses in the statistical software. Consider the following examples:
  - **Analytic code used to conduct the analyses presented in this study are available in a public archive:**  https://osf.io/hyntr/.
  - Analytic code used to conduct the analyses presented in this study are not available in a public archive. They may be available by emailing the corresponding author.
  - There is not analytic code associated with this study.
5. **Materials availability.** Materials include any stimuli used in this study, intervention protocols, and survey instruments and items. Consider the following examples:
  - **All materials used to conduct the a study are available in a public archive:**  https://osf.io/hyntr/.
  - Some of the materials used to conduct the study are presented in a public archive: ____________ (*provide url*).
  - Materials used to conduct the study are not publically available.
