# Supplementary material for: Effects of physical activity on sleep in an ecologically valid design
Source: Ann Behav Med. 2026 May 27;60(1):kaag025. doi: 10.1093/abm/kaag025 (PMC13215084; doi:10.1093/abm/kaag025)
Supplement: kaag025_Supplementary_Data [file kaag025_supplementary_data.zip › Supplementary_figures.docx]

**
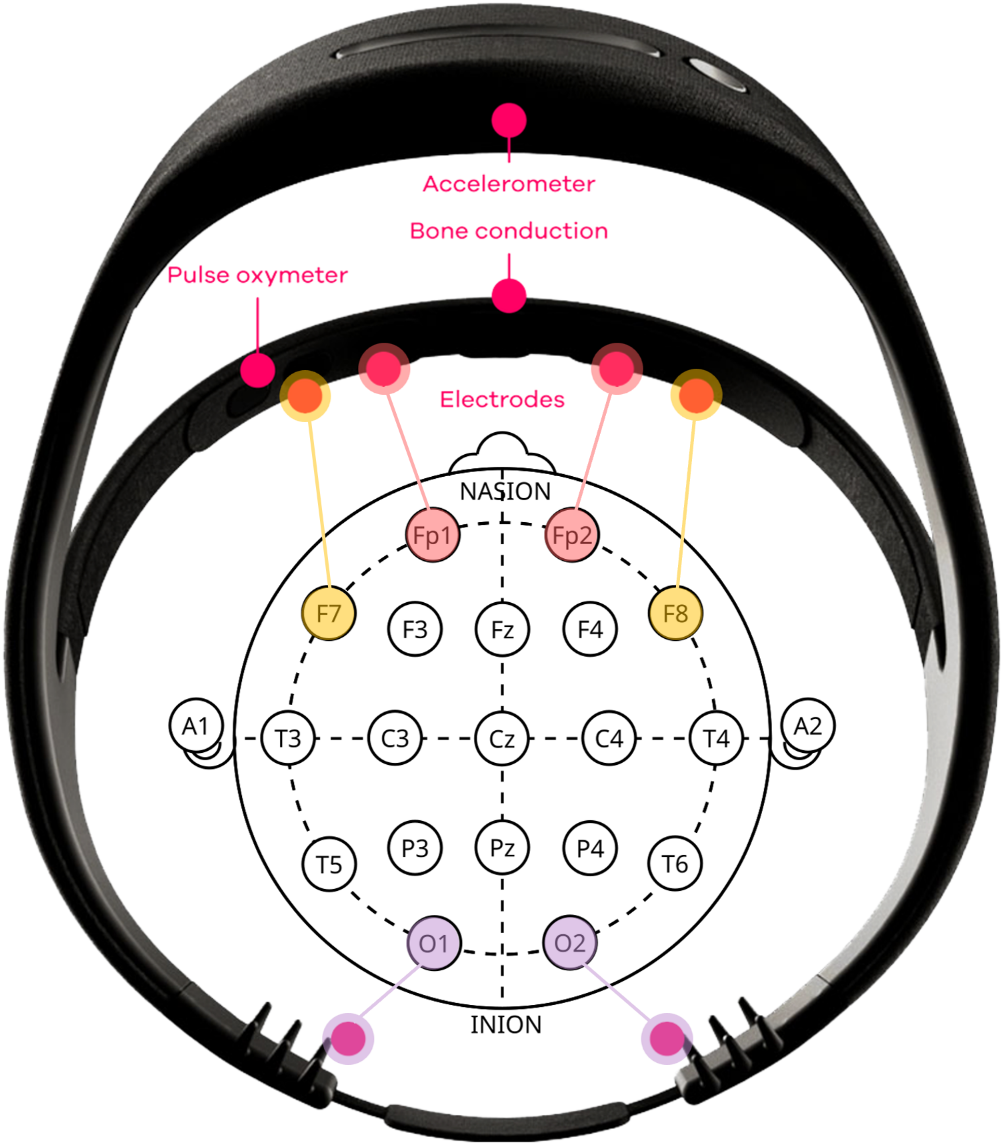
**

**Supplementary figure S1**. An illustration of the Dreem2 mobile EEG headband’s structure and its electrode placement, including a topographical representation of the electrode layout and the electrodes’ approximate positions according to the international 10-20 system. In our present study, the F7-O1 channel was used for power spectral density analyses.


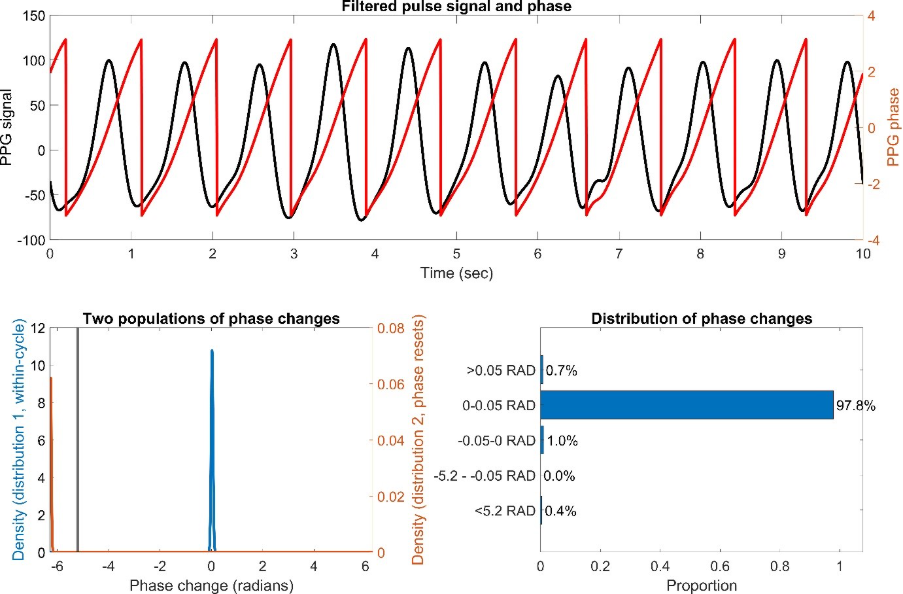


**Supplementary figure S2**. An illustration of heart rate detection from the PPG signals of the Dreem pulse oximeter. Top: raw PPG signal (black) and instantaneous phases calculated from the phase angle of the Hilbert transform (red) in 10 seconds of data from a randomly selected participant. Note that phase resets once per cardiac cycle are uniquely large datapoint-to-datapoint changes in instantaneous phase, representing a marker for detecting the heart rate. Bottom left: modelling the histogram of datapoint-to-datapoint phase changes as a mixture of two populations indeed identifies two distinct Gaussians: one with a mean slightly above 0 (within-phase changes) and one with a mean below -6 radians (phase resets). The data represents is based on all 7292250 PPG samples recorded from this participant. Note the different scale of the two histograms as phase resets are much less common than within-phase changes. Bottom right: zooming in on the histogram. Most PPG datapoints have an instantaneous phase slightly higher than the previous one. Small negative changes (~1%) represent artifacts in the PPG signal. Phase changes below -0.05 radians are completely absent, except for the 0.04% of data points below -5.2 radians which is the value at the intersection of the two fitted Gaussians in this participant. These represent phase resets. Note that with a sampling rate of 250 Hz and assuming a heart rate of 60 bpm, the expected datapoint-to-datapoint change of the PPG phase would be ~0.025 radians (2 π/250) within the cardiac cycle and 0.4% of data points (one out of the 250 representing a second) would represent a phase reset.


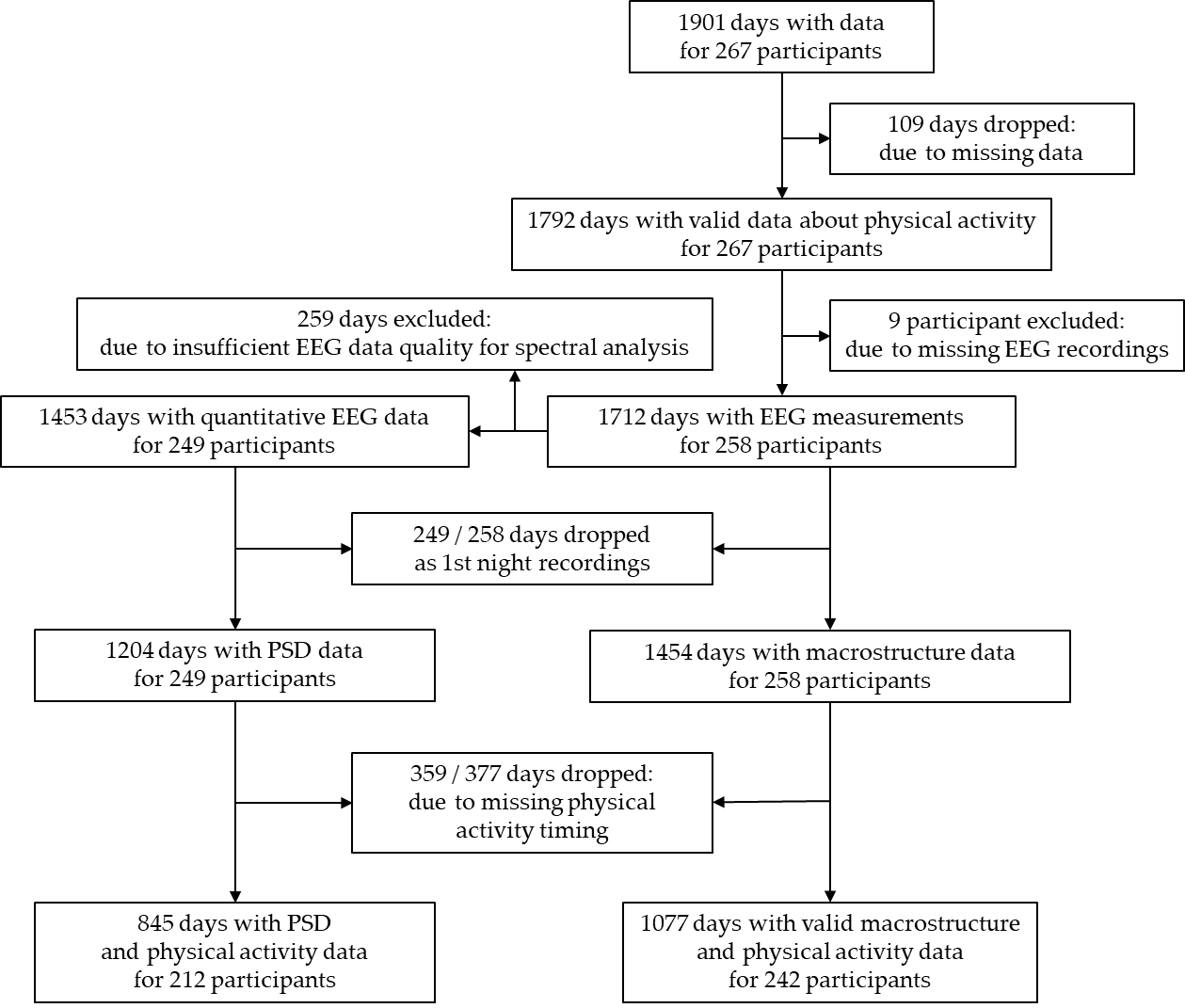
**Supplementary figure S3**. Flowchart of sample inclusion and exclusion criteria. The BSETS database contained 1901 study days from 267 participants. Of these, 1792 days included valid data about physical activity (see Table 1). Objective sleep data was unavailable from 9 participants due to non-compliance with EEG recordings or data quality insufficient even for hypnogram scoring. As described in the BSETS protocol, some recordings had sufficient data quality for hypnogram scoring, but (upon visual inspection of the spectra) not for quantitative EEG analyses (power spectral density), resulting in lower sample size in the case of the latter. Because our analyses used lagged sleep metrics from the previous night and exercise timing if exercise was reported, first-night recordings and days with data on exercise but not exercise timing were excluded.
