# Supplementary material for: Effects of physical activity on sleep in an ecologically valid design
Source: Ann Behav Med. 2026 May 27;60(1):kaag025. doi: 10.1093/abm/kaag025 (PMC13215084; doi:10.1093/abm/kaag025)
Supplement: kaag025_Supplementary_Data [file kaag025_supplementary_data.zip › Supplementary_tables.docx]

| **Study** | **k** | **N** | **Mean N** | **Significant / all effects** | **Exercise** | **Sample** | **Design** | **Hetero-geneity (%)** | **Publication bias** | **Findings** |
| --- | --- | --- | --- | --- | --- | --- | --- | --- | --- | --- |
| Amiri et al. (2021) | 32 | 1797 | 56 | 4/5 | Chronic | Non-clinical | RCT | 0-89 | none | improved sleep quality, reduced insomnia and decreased sleepiness |
| Xie et al. (2021) | 22 | 1806 | 82 | 4/7 | Chronic | Clinical | RCT | 0-70 | none | improved sleep quality (PSQI) and sleep efficiency, reduced insomnia and decreased daytime sleepiness |
| Atoui et al. (2021) | 33 | 14387 | 436 | 4/7 | Acute | Both | Any | *-* | not checked | no strong bidirectional effect |
| Frimpong et al. (2021) | 15 | 194 | 13 | 1/12 | Acute | Non-clinical | Any | 0-66 | for some outcomes, not corrected | High Intensity Exercise 4 hour before sleep had no effect except REM suppression |
| Stutz et al (2019) | 23 | 275 | 12 | 3/12 | Acute | Non-clinical | Any | 0-89 | none | intensive exercise less than 1 hour before sleep impaired sleep efficiency, decreased sleep onset latency and total sleep time; evening exercise increased REM latency and slow wave sleep, while reduced N1 sleep |
| Kredlow et al. (2015) | 41 | 1712 | 42 | 6/12 | Acute | Both | Any | * | yes, controlled | improved sleep efficiency, increased total sleep time and slow wave sleep, decreased sleep onset latency, wake after sleep onset and REM |
|  | 25 | 1151 | 46 | 4/12 | Chronic |  |  |  |  | improved sleep quality and sleep efficiency, increased total sleep time, decreased sleep onset latency |
| Youngstedt et al. (1997) | 38 | 401 | 11 | 5/7 | Acute | Non-clinical | Any | 0-45 | not checked | increased total sleep time, slow wave sleep, N2 and REM latency, decreased REM |
| Kubitz et al. (1996) | 32 | 828 | 26 | 7/20 | Acute | Non-clinical | Any | * | not checked | increased total sleep time and slow wave sleep, decreased sleep onset latency and REM |
|  | 12 | 274 | 22 | 5/18 | Chronic |  |  |  |  | increased total sleep time and slow wave sleep, decreased sleep onset latency and REM |

**Supplementary table S1.** Meta-analyses of interventional studies about the effects of physical activity on sleep. k: number of studies. N: number of participants. The table indicates the number of statistically significant effects as a proportion of all investigated effects, the type of exercise (acute: sleep changes immediately after exercise; chronic: sleep changes after a multiday training regimen), the sampling method (clinical, non-clinical, or mixed samples), statistical design (RCT only or any intervention), heterogeneity (I^2^), and publication bias. A short summary of meta-analytic findings is included. * Insufficient data is reported to quantify heterogeneity.

|  | **Exercise main effects** | | | | | | **Timing interactions** | | | | | |
| --- | --- | --- | --- | --- | --- | --- | --- | --- | --- | --- | --- | --- |
|  | **Within-participant** | | | **Between-participant** | | | **Within-participant** | | | **Between-participant** | | |
|  | ***B*** | ***SE*** | ***p*** | ***B*** | ***SE*** | ***p*** | ***B*** | ***SE*** | ***p*** | ***B*** | ***SE*** | ***p*** |
| **Total sleep time** | -18.016 | 8.040 | 0.025 | 29.392 | 13.345 | 0.028 | -0.522 | 1.854 | 0.778 | -2.310 | 4.033 | 0.567 |
| **Sleep onset latency** | -0.013 | 0.027 | 0.636 | 0.102 | 0.055 | 0.064 | 0.007 | 0.006 | 0.218 | -0.011 | 0.017 | 0.500 |
| **REM latency** | 3.312 | 3.285 | 0.314 | 9.042 | 5.430 | 0.096 | **-2.291** | **0.757** | **0.003** | 0.508 | 1.644 | 0.757 |
| **Wake after sleep onset** | -0.029 | 0.027 | 0.280 | -0.110 | 0.052 | 0.033 | 0.001 | 0.006 | 0.810 | -0.008 | 0.016 | 0.588 |
| **Sleep efficiency** | 0.000 | 0.021 | 0.994 | -0.049 | 0.046 | 0.288 | -0.002 | 0.005 | 0.692 | -0.007 | 0.014 | 0.590 |
| **N1 %** | -0.018 | 0.192 | 0.927 | -0.934 | 0.368 | 0.011 | 0.034 | 0.043 | 0.440 | 0.095 | 0.111 | 0.391 |
| **N2 %** | -0.524 | 0.706 | 0.458 | 0.951 | 1.381 | 0.491 | -0.274 | 0.160 | 0.087 | -0.579 | 0.416 | 0.164 |
| **N3 %** | 0.799 | 0.737 | 0.278 | 0.999 | 1.350 | 0.459 | -0.050 | 0.168 | 0.768 | 0.385 | 0.407 | 0.345 |
| **REM %** | -0.138 | 0.623 | 0.825 | -0.897 | 1.012 | 0.375 | 0.324 | 0.144 | 0.025 | 0.061 | 0.307 | 0.843 |
| **N1 duration** | -1.164 | 0.831 | 0.162 | -2.183 | 1.695 | 0.198 | -0.061 | 0.187 | 0.743 | 0.362 | 0.510 | 0.478 |
| **N2 duration** | -11.607 | 4.955 | 0.019 | 17.536 | 9.693 | 0.071 | -1.398 | 1.120 | 0.212 | -3.572 | 2.919 | 0.221 |
| **N3 duration** | 0.202 | 2.274 | 0.929 | 9.385 | 5.174 | 0.070 | -0.204 | 0.507 | 0.688 | 0.616 | 1.555 | 0.692 |
| **REM duration** | -6.048 | 3.442 | 0.079 | 2.715 | 5.471 | 0.620 | 1.349 | 0.799 | 0.092 | -0.099 | 1.660 | 0.952 |
| **Awakenings** | -1.468 | 0.747 | 0.050 | -3.601 | 1.632 | 0.028 | -0.017 | 0.167 | 0.920 | 0.197 | 0.490 | 0.687 |
| **Subjective sleep quality (GSQS)** | 0.264 | 0.305 | 0.386 | 0.072 | 0.498 | 0.884 | 0.103 | 0.069 | 0.137 | 0.227 | 0.150 | 0.131 |
| **Delta power** | 0.009 | 0.015 | 0.553 | 0.000 | 0.029 | 0.996 | 0.002 | 0.004 | 0.668 | -0.004 | 0.008 | 0.652 |
| **Sigma power** | -0.008 | 0.010 | 0.429 | -0.034 | 0.013 | 0.011 | 0.002 | 0.002 | 0.393 | -0.004 | 0.004 | 0.263 |
| **Respiration rate** | -0.011 | 0.100 | 0.914 | 0.116 | 0.131 | 0.376 | 0.024 | 0.024 | 0.307 | -0.056 | 0.040 | 0.166 |
| **Heart rate (wake)** | 0.548 | 0.514 | 0.287 | **-4.841** | **1.297** | **<0.001** | -0.055 | 0.111 | 0.621 | -0.449 | 0.383 | 0.242 |
| **Heart rate variability (wake)** | -0.643 | 9.491 | 0.946 | -19.842 | 20.255 | 0.328 | 0.497 | 2.080 | 0.811 | -3.538 | 5.996 | 0.555 |
| **Heart rate (1st hour)** | 0.930 | 0.711 | 0.191 | **-5.934** | **1.560** | **<0.001** | -0.077 | 0.156 | 0.619 | -0.611 | 0.459 | 0.183 |
| **Heart rate variability (1st hour)** | 20.070 | 14.641 | 0.171 | -61.905 | 27.790 | 0.026 | -3.702 | 3.258 | 0.256 | -6.509 | 8.194 | 0.427 |

**Supplementary table S2**. The effects of exercise and its timing on sleep variables. Regression coefficients indicate the estimated changes in sleep parameters after exercise (main effects), or changes in these effects per one hour of additional distance between exercise midpoint and sleep (interactions). Within-participant effects indicate sleep changes within the same participant after exercise, while between-participant effects are correlations of exercise habits and average sleep. All regression coefficients are expressed in raw units, and in minutes in case of timing variables. Bold values indicate effects which survive correction for multiple comparisons. Values in italics do not survive this correction but are putatively significant at p<0.01.

|  | **Exercise main effects** | | | | | | **Timing interactions** | | | | | |
| --- | --- | --- | --- | --- | --- | --- | --- | --- | --- | --- | --- | --- |
|  | **Within-participant** | | | **Between-participant** | | | **Within-participant** | | | **Between-participant** | | |
|  | ***B*** | ***SE*** | ***p*** | ***B*** | ***SE*** | ***p*** | ***B*** | ***SE*** | ***p*** | ***B*** | ***SE*** | ***p*** |
| **Total sleep time** | -16.222 | 7.976 | 0.042 | 30.026 | 13.324 | 0.024 | -1.375 | 1.857 | 0.459 | -1.856 | 4.084 | 0.650 |
| **Sleep onset latency** | -0.014 | 0.027 | 0.592 | 0.102 | 0.055 | 0.063 | 0.007 | 0.006 | 0.254 | -0.011 | 0.017 | 0.519 |
| **REM latency** | 2.464 | 3.252 | 0.449 | 8.769 | 5.399 | 0.105 | **-2.730** | **0.758** | **<0.001** | 0.906 | 1.658 | 0.585 |
| **Wake after sleep onset** | -0.035 | 0.026 | 0.190 | -0.112 | 0.052 | 0.031 | 0.000 | 0.006 | 0.961 | -0.009 | 0.016 | 0.550 |
| **Sleep efficiency** | -0.006 | 0.021 | 0.777 | -0.050 | 0.046 | 0.272 | -0.002 | 0.005 | 0.661 | -0.008 | 0.014 | 0.580 |
| **N1 %** | -0.028 | 0.190 | 0.883 | -0.941 | 0.369 | 0.011 | 0.032 | 0.043 | 0.459 | 0.107 | 0.112 | 0.340 |
| **N2 %** | -0.520 | 0.698 | 0.457 | 0.975 | 1.381 | 0.480 | -0.329 | 0.160 | 0.039 | -0.555 | 0.422 | 0.188 |
| **N3 %** | 0.798 | 0.730 | 0.274 | 0.972 | 1.351 | 0.472 | 0.022 | 0.168 | 0.894 | 0.301 | 0.413 | 0.467 |
| **REM %** | -0.138 | 0.616 | 0.823 | -0.890 | 1.014 | 0.380 | 0.305 | 0.144 | 0.034 | 0.108 | 0.312 | 0.728 |
| **N1 duration** | -1.111 | 0.822 | 0.177 | -2.157 | 1.686 | 0.201 | -0.107 | 0.187 | 0.567 | 0.451 | 0.514 | 0.381 |
| **N2 duration** | -10.269 | 4.924 | 0.037 | 18.021 | 9.665 | 0.063 | -1.896 | 1.126 | 0.092 | -3.311 | 2.947 | 0.261 |
| **N3 duration** | 0.168 | 2.246 | 0.940 | 9.340 | 5.186 | 0.072 | -0.152 | 0.506 | 0.764 | 0.373 | 1.576 | 0.813 |
| **REM duration** | -5.578 | 3.411 | 0.102 | 2.930 | 5.476 | 0.593 | 1.026 | 0.799 | 0.199 | 0.205 | 1.686 | 0.903 |
| **Awakenings** | -1.528 | 0.738 | 0.039 | -3.643 | 1.637 | 0.026 | -0.054 | 0.167 | 0.747 | 0.229 | 0.497 | 0.646 |
| **Subjective sleep quality (GSQS)** | 0.350 | 0.303 | 0.247 | 0.086 | 0.494 | 0.862 | 0.104 | 0.069 | 0.134 | 0.211 | 0.152 | 0.165 |
| **Delta power** | 0.010 | 0.015 | 0.488 | 0.001 | 0.029 | 0.984 | 0.002 | 0.004 | 0.541 | -0.007 | 0.008 | 0.427 |
| **Sigma power** | -0.008 | 0.010 | 0.441 | -0.034 | 0.013 | 0.010 | 0.003 | 0.002 | 0.307 | -0.006 | 0.004 | 0.129 |
| **Respiration rate** | -0.012 | 0.099 | 0.900 | 0.118 | 0.131 | 0.367 | 0.021 | 0.024 | 0.385 | -0.055 | 0.041 | 0.184 |
| **Heart rate (wake)** | 0.572 | 0.510 | 0.262 | **-4.802** | **1.297** | **<0.001** | -0.079 | 0.112 | 0.480 | -0.357 | 0.387 | 0.357 |
| **Heart rate variability (wake)** | 1.755 | 9.441 | 0.853 | -18.836 | 20.191 | 0.351 | 0.158 | 2.094 | 0.940 | -3.192 | 6.043 | 0.597 |
| **Heart rate (1st hour)** | 0.986 | 0.705 | 0.162 | **-5.872** | **1.558** | **<0.001** | -0.114 | 0.156 | 0.467 | -0.516 | 0.463 | 0.266 |
| **Heart rate variability (1st hour)** | 21.791 | 14.528 | 0.134 | -61.020 | 27.798 | 0.028 | -3.659 | 3.268 | 0.263 | -5.574 | 8.291 | 0.502 |

**Supplementary table S3**. The effects of exercise and its timing on sleep variables. Regression coefficients indicate the estimated changes in sleep parameters after exercise (main effects), or changes in these effects per one hour of additional distance between exercise end time and sleep (interactions). Within-participant effects indicate sleep changes within the same participant after exercise, while between-participant effects are correlations of exercise habits and average sleep. All regression coefficients are expressed in raw units, and in minutes in case of timing variables. Bold values indicate effects which survive correction for multiple comparisons. Values in italics do not survive this correction but are putatively significant at p<0.01.

|  | **Exercise main effects** | | | | | | **Timing of exercise** | | | | | |
| --- | --- | --- | --- | --- | --- | --- | --- | --- | --- | --- | --- | --- |
|  | **Within-participant** | | | **Between-participant** | | | **Within-participant** | | | **Between-participant** | | |
|  | ***B*** | ***SE*** | ***p*** | ***B*** | ***SE*** | ***p*** | ***B*** | ***SE*** | ***p*** | ***B*** | ***SE*** | ***p*** |
| **Total sleep time** | -8.374 | 7.035 | 0.234 | 30.407 | 12.423 | 0.015 | -0.483 | 1.388 | 0.728 | 0.373 | 2.510 | 0.882 |
| **Sleep onset latency** | -0.020 | 0.023 | 0.380 | 0.098 | 0.052 | 0.062 | 0.003 | 0.005 | 0.492 | -0.007 | 0.011 | 0.497 |
| **REM latency** | 3.721 | 2.847 | 0.192 | 8.822 | 5.308 | 0.097 | *1.594* | *0.558* | *0.004* | 1.063 | 1.075 | 0.323 |
| **Wake after sleep onset** | -0.017 | 0.023 | 0.468 | -0.076 | 0.050 | 0.130 | -0.009 | 0.005 | 0.060 | -0.011 | 0.010 | 0.289 |
| **Sleep efficiency** | -0.011 | 0.018 | 0.564 | -0.038 | 0.044 | 0.395 | -0.001 | 0.004 | 0.839 | -0.014 | 0.009 | 0.127 |
| **N1 %** | 0.079 | 0.164 | 0.629 | -0.929 | 0.377 | 0.014 | -0.046 | 0.032 | 0.148 | -0.006 | 0.077 | 0.940 |
| **N2 %** | 0.181 | 0.609 | 0.766 | 0.950 | 1.360 | 0.485 | 0.154 | 0.118 | 0.192 | -0.140 | 0.277 | 0.613 |
| **N3 %** | 0.184 | 0.638 | 0.773 | 0.521 | 1.264 | 0.681 | 0.113 | 0.125 | 0.364 | 0.026 | 0.257 | 0.918 |
| **REM %** | -0.322 | 0.546 | 0.555 | -0.454 | 0.946 | 0.632 | -0.228 | 0.108 | 0.035 | 0.169 | 0.192 | 0.377 |
| **N1 duration** | -0.171 | 0.706 | 0.808 | -2.058 | 1.766 | 0.244 | -0.103 | 0.137 | 0.454 | 0.018 | 0.360 | 0.961 |
| **N2 duration** | -3.882 | 4.348 | 0.372 | 19.136 | 9.307 | 0.040 | 0.455 | 0.849 | 0.592 | -0.893 | 1.890 | 0.637 |
| **N3 duration** | -0.201 | 1.942 | 0.917 | 8.505 | 5.124 | 0.097 | 0.220 | 0.375 | 0.558 | 0.735 | 1.044 | 0.482 |
| **REM duration** | -4.449 | 3.004 | 0.139 | 4.163 | 4.972 | 0.403 | -1.112 | 0.595 | 0.062 | 0.849 | 1.006 | 0.399 |
| **Awakenings** | -0.764 | 0.628 | 0.224 | -3.303 | 1.642 | 0.045 | -0.058 | 0.121 | 0.636 | -0.091 | 0.334 | 0.786 |
| **Subjective sleep quality (GSQS)** | 0.219 | 0.262 | 0.402 | 0.214 | 0.459 | 0.642 | -0.022 | 0.051 | 0.669 | -0.182 | 0.093 | 0.050 |
| **Delta power** | -0.007 | 0.013 | 0.595 | -0.013 | 0.027 | 0.639 | 0.000 | 0.003 | 0.867 | -0.001 | 0.007 | 0.911 |
| **Sigma power** | -0.006 | 0.009 | 0.473 | **-0.032** | **0.012** | **0.007** | 0.000 | 0.002 | 0.966 | -0.001 | 0.003 | 0.631 |
| **Respiration rate** | -0.007 | 0.086 | 0.935 | 0.157 | 0.115 | 0.173 | -0.014 | 0.017 | 0.429 | 0.030 | 0.023 | 0.196 |
| **Heart rate (wake)** | 0.591 | 0.431 | 0.170 | **-4.178** | **1.242** | **0.001** | 0.167 | 0.083 | 0.045 | -0.121 | 0.260 | 0.643 |
| **Heart rate variability (wake)** | -3.245 | 7.880 | 0.681 | -22.233 | 19.146 | 0.246 | -0.181 | 1.530 | 0.906 | -3.916 | 4.026 | 0.331 |
| **Heart rate (1st hour)** | 0.990 | 0.602 | 0.100 | **-5.572** | **1.457** | **<0.001** | 0.232 | 0.117 | 0.048 | -0.088 | 0.304 | 0.773 |
| **Heart rate variability (1st hour)** | 12.147 | 12.258 | 0.322 | **-71.801** | **26.150** | **0.006** | 1.283 | 2.393 | 0.592 | 0.742 | 5.488 | 0.892 |

**Supplementary table S4.** The effects of exercise and its clock time (instead of relative timing compared to sleep as in the main analysis) on sleep variables. Regression coefficients indicate the estimated changes in sleep parameters after exercise (main effects), or changes in these effects per one hour exercise clock time (interactions). Within-participant effects indicate sleep changes within the same participant after exercise, while between-participant effects are correlations of exercise habits and average sleep. All regression coefficients are expressed in raw units, and in minutes in case of timing variables. Bold values indicate effects which survive correction for multiple comparisons. Values in italics do not survive this correction but are putatively significant at p<0.01.

|  | **Morning** | | | **Afternoon** | | | **Evening** | | |
| --- | --- | --- | --- | --- | --- | --- | --- | --- | --- |
|  | ***B*** | ***SE*** | ***p*** | ***B*** | ***SE*** | ***p*** | ***B*** | ***SE*** | ***p*** |
| **Total sleep time** | -1.094 | 29.594 | 0.971 | -6.350 | 18.809 | 0.736 | 5.860 | 19.546 | 0.765 |
| **Sleep onset latency** | -0.086 | 0.093 | 0.354 | 0.079 | 0.059 | 0.184 | 0.026 | 0.061 | 0.677 |
| **REM latency** | -1.280 | 13.566 | 0.925 | 0.387 | 8.623 | 0.964 | 6.060 | 8.963 | 0.500 |
| **Wake after sleep onset** | 0.126 | 0.104 | 0.228 | -0.088 | 0.066 | 0.183 | 0.020 | 0.069 | 0.773 |
| **Sleep efficiency** | -0.012 | 0.078 | 0.874 | 0.012 | 0.050 | 0.807 | 0.011 | 0.052 | 0.838 |
| **N1 %** | 0.391 | 0.784 | 0.619 | -0.905 | 0.499 | 0.071 | -0.498 | 0.518 | 0.337 |
| **N2 %** | 2.937 | 2.700 | 0.278 | -1.784 | 1.716 | 0.300 | 1.358 | 1.784 | 0.447 |
| **N3 %** | -1.123 | 2.955 | 0.704 | 4.483 | 1.878 | 0.018 | -1.472 | 1.952 | 0.452 |
| **REM %** | -2.204 | 2.408 | 0.361 | -1.795 | 1.531 | 0.242 | 0.613 | 1.591 | 0.700 |
| **N1 duration** | 2.572 | 3.202 | 0.423 | -2.506 | 2.035 | 0.219 | -0.969 | 2.115 | 0.647 |
| **N2 duration** | 9.026 | 18.602 | 0.628 | -11.540 | 11.823 | 0.330 | 9.817 | 12.286 | 0.425 |
| **N3 duration** | -2.286 | 9.189 | 0.804 | *15.754* | *5.840* | *0.008* | -4.742 | 6.069 | 0.435 |
| **REM duration** | -10.406 | 11.918 | 0.384 | -8.058 | 7.575 | 0.289 | 1.754 | 7.872 | 0.824 |
| **Awakenings** | 0.084 | 2.680 | 0.975 | -1.690 | 1.703 | 0.322 | -0.903 | 1.770 | 0.611 |
| **Subjective sleep quality (GSQS)** | 0.065 | 1.005 | 0.949 | -1.104 | 0.663 | 0.097 | -0.845 | 0.684 | 0.218 |
| **Delta power** | 0.010 | 0.054 | 0.857 | 0.014 | 0.033 | 0.668 | -0.043 | 0.037 | 0.245 |
| **Sigma power** | -0.008 | 0.045 | 0.853 | -0.038 | 0.028 | 0.174 | -0.007 | 0.031 | 0.813 |
| **Respiration rate** | 1.109 | 0.957 | 0.248 | 1.060 | 0.608 | 0.083 | 1.440 | 0.632 | 0.024 |
| **Heart rate (wake)** | -1.159 | 3.411 | 0.734 | 4.524 | 2.225 | 0.043 | -2.279 | 2.180 | 0.297 |
| **Heart rate variability (wake)** | -51.963 | 56.156 | 0.356 | *98.298* | *36.627* | *0.008* | -2.209 | 35.899 | 0.951 |
| **Heart rate (1st hour)** | 0.091 | 3.611 | 0.980 | 4.051 | 2.355 | 0.087 | -3.805 | 2.309 | 0.101 |
| **Heart rate variability (1st hour)** | -44.654 | 71.452 | 0.533 | 84.737 | 46.604 | 0.071 | -50.254 | 45.677 | 0.273 |

**Supplementary table S5**. The effects of exercise on sleep variables categorized by timing. Regression coefficients indicate the estimated changes in sleep parameters after exercise. All regression coefficients are expressed in raw units, and in minutes in case of timing variables. Values in italics do not survive correction for multiple comparisons but are putatively significant at p<0.01.

|  | **Exercise main effects** | | | | | |
| --- | --- | --- | --- | --- | --- | --- |
|  | **Within-participant** | | | **Between-participant** | | |
|  | ***B*** | ***SE*** | ***p*** | ***B*** | ***SE*** | ***p*** |
| **Total sleep time** | -6.693 | 5.872 | 0.255 | 19.102 | 11.292 | 0.091 |
| **Sleep onset latency** | -0.018 | 0.019 | 0.334 | 0.108 | 0.050 | 0.030 |
| **REM latency** | 2.580 | 2.286 | 0.259 | 4.746 | 4.949 | 0.338 |
| **Wake after sleep onset** | -0.023 | 0.019 | 0.212 | -0.054 | 0.049 | 0.271 |
| **Sleep efficiency** | -0.013 | 0.015 | 0.385 | 0.008 | 0.043 | 0.846 |
| **N1 %** | -0.100 | 0.128 | 0.436 | **-0.981** | **0.371** | **0.008** |
| **N2 %** | -0.371 | 0.498 | 0.456 | 0.642 | 1.333 | 0.630 |
| **N3 %** | 0.663 | 0.511 | 0.195 | 0.535 | 1.229 | 0.664 |
| **REM %** | -0.175 | 0.464 | 0.707 | -0.136 | 0.923 | 0.883 |
| **N1 duration** | -0.626 | 0.563 | 0.266 | -2.519 | 1.676 | 0.133 |
| **N2 duration** | -4.123 | 3.576 | 0.249 | 14.911 | 8.783 | 0.090 |
| **N3 duration** | 1.287 | 1.557 | 0.409 | 5.944 | 4.727 | 0.209 |
| **REM duration** | -3.252 | 2.476 | 0.189 | 1.455 | 4.448 | 0.744 |
| **Awakenings** | -0.759 | 0.496 | 0.126 | -3.438 | 1.582 | 0.030 |
| **Subjective sleep quality (GSQS)** | 0.227 | 0.214 | 0.288 | 0.418 | 0.417 | 0.316 |
| **Delta power** | -0.001 | 0.010 | 0.906 | 0.009 | 0.029 | 0.763 |
| **Sigma power** | -0.004 | 0.006 | 0.503 | -0.039 | 0.024 | 0.101 |
| **Respiration rate** | -0.019 | 0.070 | 0.783 | 0.145 | 0.098 | 0.140 |
| **Heart rate (wake)** | 0.519 | 0.348 | 0.137 | **-4.231** | **1.179** | **<0.001** |
| **Heart rate variability (wake)** | 4.767 | 6.389 | 0.456 | -20.390 | 17.975 | 0.257 |
| **Heart rate (1st hour)** | 0.854 | 0.482 | 0.076 | **-5.884** | **1.408** | **<0.001** |
| **Heart rate variability (1st hour)** | 18.427 | 10.531 | 0.080 | **-73.370** | **24.163** | **0.002** |

**Supplementary table S6.** The effects of exercise on sleep variables. Regression coefficients indicate the estimated changes in sleep parameters after exercise (main effects). Within-participant effects indicate sleep changes within the same participant after exercise, while between-participant effects are correlations of exercise habits and average sleep. All regression coefficients are expressed in raw units, and in minutes in case of timing variables. Bold values indicate effects which survive correction for multiple comparisons. Values in italics do not survive this correction but are putatively significant at p<0.01.

|  | **Physical exhaustion effects** | | | | | |
| --- | --- | --- | --- | --- | --- | --- |
|  | **Within-participant** | | | **Between-participant** | | |
|  | ***B*** | ***SE*** | ***p*** | ***B*** | ***SE*** | ***p*** |
| **Total sleep time** | -0.242 | 1.146 | 0.833 | -0.946 | 2.071 | 0.648 |
| **Sleep onset latency** | -0.008 | 0.004 | 0.021 | -0.015 | 0.009 | 0.096 |
| **REM latency** | 1.007 | 0.446 | 0.024 | 0.003 | 0.914 | 0.997 |
| **Wake after sleep onset** | 0.000 | 0.004 | 0.918 | -0.016 | 0.009 | 0.080 |
| **Sleep efficiency** | -0.005 | 0.003 | 0.109 | -0.015 | 0.008 | 0.048 |
| **N1 %** | 0.018 | 0.025 | 0.483 | -0.122 | 0.069 | 0.076 |
| **N2 %** | 0.167 | 0.097 | 0.085 | -0.169 | 0.245 | 0.491 |
| **N3 %** | -0.037 | 0.100 | 0.711 | 0.456 | 0.225 | 0.043 |
| **REM %** | -0.161 | 0.090 | 0.075 | -0.152 | 0.173 | 0.380 |
| **N1 duration** | 0.045 | 0.110 | 0.681 | -0.436 | 0.307 | 0.156 |
| **N2 duration** | 0.359 | 0.699 | 0.607 | -1.176 | 1.629 | 0.470 |
| **N3 duration** | -0.092 | 0.303 | 0.760 | 1.932 | 0.865 | 0.026 |
| **REM duration** | -0.561 | 0.483 | 0.245 | -0.974 | 0.816 | 0.233 |
| **Awakenings** | -0.040 | 0.097 | 0.679 | -0.284 | 0.293 | 0.333 |
| **Subjective sleep quality (GSQS)** | 0.001 | 0.042 | 0.978 | 0.097 | 0.074 | 0.191 |
| **Delta power** | 0.001 | 0.002 | 0.657 | 0.008 | 0.005 | 0.125 |
| **Sigma power** | -0.001 | 0.001 | 0.559 | 0.004 | 0.005 | 0.325 |
| **Respiration rate** | 0.033 | 0.014 | 0.017 | 0.020 | 0.018 | 0.266 |
| **Heart rate (wake)** | **0.381** | **0.068** | **<0.001** | -0.187 | 0.212 | 0.378 |
| **Heart rate variability (wake)** | 1.121 | 1.270 | 0.378 | -1.059 | 3.188 | 0.740 |
| **Heart rate (1st hour)** | **0.458** | **0.095** | **<0.001** | -0.253 | 0.256 | 0.322 |
| **Heart rate variability (1st hour)** | 3.493 | 2.093 | 0.095 | 0.115 | 4.322 | 0.979 |

**Supplementary table S7.** The effects of self-reported physical exhaustion on sleep variables. Regression coefficients indicate the estimated changes in sleep parameters per one-unit increase in subjective physical exhaustion rating. Within-participant effects indicate sleep changes within the same participant after exercise, while between-participant effects are correlations of exercise habits and average sleep. All regression coefficients are expressed in raw units, and in minutes in case of timing variables. Bold values indicate effects which survive correction for multiple comparisons. Values in italics do not survive this correction but are putatively significant at p<0.01.
